# Serum pepsinogen II levels are doubled with *Helicobacter pylori* infection in an asymptomatic population of 40,383 Chinese subjects

**DOI:** 10.1097/MD.0000000000026562

**Published:** 2021-07-09

**Authors:** Hong Yu, Ying Liu, Shujing Jiang, Yunfeng Zhou, Zheng Guan, Siyuan Dong, Fong-Fong Chu, Chunbo Kang, Qiang Gao

**Affiliations:** a Center of Health Management, Beijing Rehabilitation Hospital, Capital Medical University, Beijing, China; b Department of Cardiology, Royal Papworth Hospital NHS Foundation Trust, Papworth Everard, Cambridge, UK; c Department of Physiology, Medical Research Center, Shenzhen University, Shenzhen, China; d Beijing Deep Intelligent Pharma Technologies Co., Ltd., Beijing, China; e Department of Cancer Genetics and Epigenetics, Beckman Research Institute of the City of Hope, Duarte, CA; f Department of Gastroenterology and Hepatology, Beijing Rehabilitation Hospital, Capital Medical University, Beijing, China.

**Keywords:** age, body mass index, sex, *Helicobacter pylori*, pepsinogen

## Abstract

Pepsinogen (PG) I and II are crucial in the gastric digestive processes. This study is to examine the relationship of serum PGI, PGII, and PGI/PGII ratio with *Helicobacter pylori* (Hp) infection, age, sex, and body mass index (BMI) in subjects in Beijing, China.

A total of 40,383 asymptomatic subjects, who underwent medical examination in Beijing Rehabilitation Hospital, were included in this study. Serum PG levels were measured using chemoluminescence techniques. The age, sex, and BMI data were collected, and Hp infection was identified with ^13^C-urea breath test. Statistical analysis was conducted with Python, Pandas and Seaborn software.

Asymptomatic subjects with Hp infection (Hp+) had a significantly higher level of PGI in the serum (111 ng/mL [median]) than those without Hp infection (Hp−) (94 ng/mL, *P* < .001). The asymptomatic Hp+ subjects had 2-fold higher PGII levels (7.2 ng/mL) than Hp− subjects (3.2 ng/mL, *P* < .001). These changes produced significantly lower PGI/II ratio in Hp+ patients than in Hp− subjects (16:30, *P* < .001). The serum PGI and PGII levels were higher in males than in females (PGI: 104 ng/mL vs 95 ng/mL, PGII: 4.3 ng/mL vs 3.7 ng/mL, both *P* < .001), PGI/II ratio of males is at 95% of that in females (*P* < .001). PGI and PGII levels gradually increased in older people (*P* < .001), whereas the PGI/II ratio decreased significantly with age (*P* < .001). The levels of the two serum PGs were decreased and the ratio increased when BMI were higher than 28 kg/cm^2^ (*P* < .05).

The levels of serum PGI, especial PGII, were increased by Hp infection, and also influenced by age, sex, and BMI. Therefore, these influencing factors should be considered during clinical practice.

## 1 Introduction

Human gastric pepsinogen I (PGI) and pepsinogen II (PGII) are synthesized, secreted and converted into the gastric digestive pepsin.^[1]^ There are plenty of physiological or external chemical signals to stimulus the secretion of PGs in the stomach, and about 1% of PGI and PGII are secreted entering into circulating blood.^[1,2]^ Their levels in blood correlate to the morphologic and functional changes in the stomach, hence are noninvasive indicators of gastric mucosal status.^[3]^ Investigations were conducted on the relationship between serum PG levels and gastric disorders, such as chronic atrophic gastritis (CAG) and gastric cancer (GC), gastric ulcers and other gastric diseases. The concentration of PGs and ratio of PGI/PGII have been proposed as biomarkers for predicting CAG or GC.^[3,4]^

Helicobacter pylori (Hp) is a Gram-negative, microaerophillic bacterium inhabited on the luminal surface of the gastric epithelium.^[5]^ Approximately half of Chinese population is infected with Hp, and 15% to 20% of the infected individuals develop clinical diseases.^[6]^ As Hp is one of the main causes for CAG and GC,^[7]^ there has been increasing interest regarding the relationship of PG concentration and Hp infection.^[8,9]^ Chronic Hp infection caused loss of chief cells which are replaced by pylori glands.^[3,10]^ Loss of chief cells results in decrease in PGI levels, and increase of pylori glands excrete more PGII. It is believed that a low serum PGI level and low PGI/PGII ratio have been associated with severe gastric atrophy, intestinal metaplasia, and are frequently found in gastric cancer.^[4,11]^

The synthesis and secretion of pepsinogens may be associated with other factors, such as sex, age, and body mass index (BMI). These influencing factors may have clinical significance. Both sex and age are an important factor which affects many physiological and pathological processes of the human stomach. Kitahara et al^[12]^ reported PGII level increased and ratio of PG I/II level decreased with progression of age. Obesity was steadily becoming a worldwide health problem; it not only had positive effect on metabolic and cardiovascular diseases but also linked to other conditions, including gastric disorders.^[13,14]^ Kim et al^[15]^ found that high BMI was associated with gastric cancer in males and with gastric dysplasia in females of Korean origin. However, only a few studies with smaller numbers of subjects were conducted to explore the characteristics of serum PG levels in Chinese people.^[16,17]^

In the present study, we analyzed the data from a sample of asymptomatic 40,383 residents from Beijing, China, who underwent medical examination. We investigated the characteristics of serum PG levels and the ratio of PGI and PGII, and their relationship with people's age, sex, BMI, and Hp-infection status. This work may provide useful reference for clinical practice.

## 2 Materials and methods

### 2.1 Subjects

**Figure 1:**
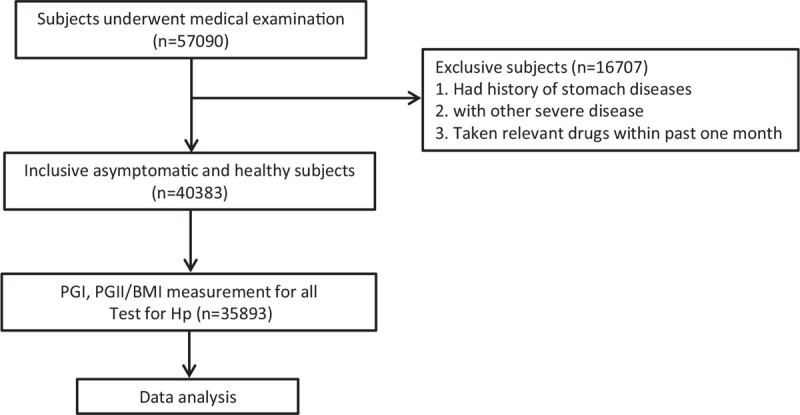
The flow chart of the study.

A total of 57,090 asymptomatic subjects who underwent medical examination in Beijing Rehabilitation Hospital from January 2016 to December 2018, were enrolled in this study, and 40,383 subjects of total number were inclusive into the study. The inclusion criteria are asymptomatic and healthy subjects. The exclusion criteria are: persons with a previous or current history of peptic ulcers, erosive gastritis, CAG or GC, and other stomach diseases; persons with a history of severe diseases of liver, kidney, heart, brain; subjects had used antibiotics, acid-suppressing drugs within the past month. Inclusive subjects had 26,259 men and 14,124 women, and their age ranged from 15 to 96 years with a mean age of 48 ± 14 years (mean ± standard deviation). They were measured for body mass index (BMI) and collected the blood samples for the measurement of PG1 and PG2, and 35,893 subjects of all individuals were tested for Hp infection using ^13^C-urea breath test (UBT) (Fig. 1). This study was approved by Hospital Ethics Committees (No 2015–012). The informed consent was obtained from all participants during the medical examination in accordance with the Declaration of Helsinki.

### 2.2 Serum pepsinogen level

Approximately 5 mL of blood was collected from each participant who had fasted overnight and kept at 4°C. Within 24 hours of the blood collection, the serum was obtained by centrifugation and the PG1 and PG2 concentrations were measured by chemoluminescence with a PGI and a PGII kits (Henan Meikai Biotechnology Co., Ltd., China). The chemoluminescence detector (TZD-200S) (Henan Meikai Biotechnology Co., Ltd., China) was used to measure the luminescence in the 96-well microtiter plates. The threshold value of the kits for PGI was >65.0 ng/mL, and PGII <15.0 ng/mL, the PGI/ PGII >7.5.

### 2.3 Identification of Hp infection

^13^C-UBT was performed using HY-IREXB ^13^C infrared spectrometer (Guangzhou Huayou Mingkang Photoelectricity Technology Co. Ltd., China) to detect urea levels. We followed the instruction of the ^13^C-UBT kit (Guangzhou Huagen Biotechnology Co.,Ltd. China). First, before ^13^C-urea administration, a breath sample was collected using a special breath collection bag to obtain a baseline value of 4.0. Then, 20 minutes after ingestion of 75 mg ^13^C-urea, a second breath sample was collected. The breath samples were analyzed using the spectrometer. An Hp+ value was >4 and Hp− value was ≤4.

### 2.4 Statistical analysis

All statistical analyses were performed using data processing software (Python Pandas package),^[18,19]^ drawing software (Python Matplotlib and Seaborn package),^[20]^ and statistical analysis software (Python Statsmodels and SciPy package).^[21]^ The value was shown as the mean ± SEM, interquartile range (IQR) (25%–75%). The student *t* test and Mann–Whitney *U* test were used in this study, a *P* value of <.05 was considered statistically significant.

## 3 Results

*PG1 and PG2 levels and PG1/PG2 ratio changes according to H pylori infection status.* From all 40,383 subjects, the serum PGI and PGII levels were 100 (73–128) ng/mL (median, 25%–75%, the same as followings) and 4.1 (2.2–7.1) ng/mL, the PGI/II ratio was 25 (15–43). Among subjects underwent UBT to determine their Hp infection status, we found 11,767 or 32.8% were positive for Hp. To investigate the correlation of Hp infection with serum PGI, PGII levels, and PGI/PGII ratio, we found that subjects with Hp+ had significantly higher PGI, PGII levels, and lower PGI/PGII ratio than Hp− subjects (*P* < .001 for all) (Table 1).

**Table 1:** Hp infection and serum PG levels (median [IQR 25%–75%]).

	N	PGI, ng/mL	PGII, ng/mL	PGI/PGII
Hp +	24126	94 (75–121)	3.2 (1.8–5.2)	30
Hp −	11767	111 (88–142)	7.2 (4.4–10.8)	16
*P* value of U test		<.001	<.001	<.001

Hp = *Helicobacter pylori*, IQR = interquartile range, PG = pepsinogen I, PGII = pepsinogen II.

**Figure 2:**
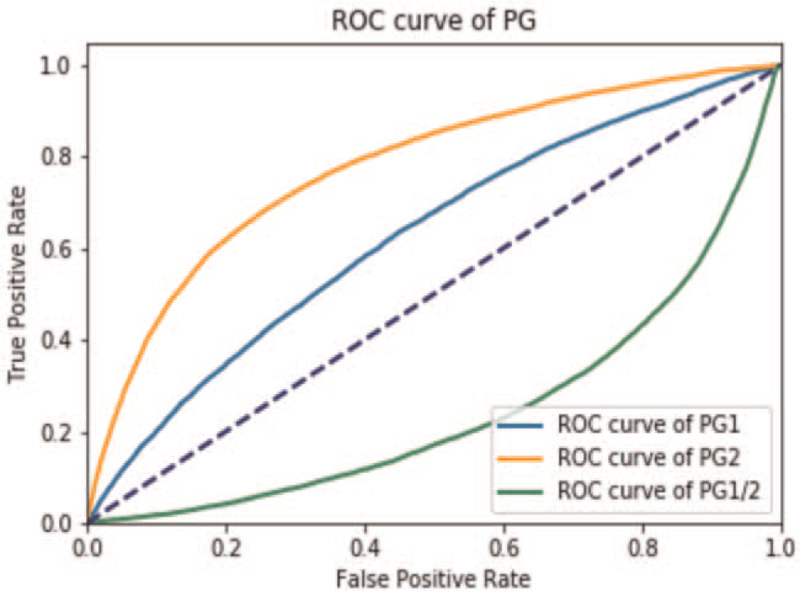
The ROC curves for PGI, PGII, and the PGI/II ratio. The areas under the ROC curves for PGI, PGII (*P* values >.5), and the PGI/II ratio (*P* values <.5) indicated that Hp infection had a positive correlation with PGI and PGII, and a negative correlation with PGI/PGII. Hp = *helicobacter pylori*, PG = pepsinogen, ROC = receiver-operating characteristic curve.

When we plot the data based on Hp− and Hp+ status and performed the Mann–Whitney *U* test on the two datasets. As shown in Figure 2 the areas under the receiver operating characteristic (ROC) curves for PGI, PGII, and the PGI/II ratio were 0.626, 0.775, and 0.248, respectively. The ROC values of PGI and PGII were >0.5, PGI/II ratio <0.5, so Hp infection had a positive correlation with PGI and PGII, and a negative correlation with PGI/PGII

### 3.1 PGI and PG2 levels were higher in male than female and increased with age

**Figure 3:**
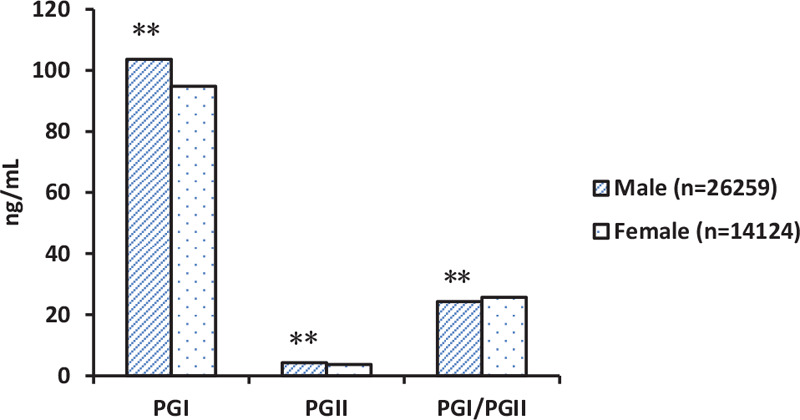
Serum PG levels in different sex. The PGI and PGII levels were higher in males than in females, whereas the PGI/II ratio was significantly lower in males than in females. Data are means ± SEM. ^∗∗^*P* < .01 compared to female. PG = pepsinogen.

Analyzed from male and female individuals, we found the PGI and PGII levels were higher in males than in females (PGI: 104 [81–133] ng/mL vs 95 [75–122] ng/mL, PGII: 4.3 [2.4–7.4] ng/mL vs 3.7 [2.0–6.7] ng/mL, both *P* < .001), whereas the PGI/II ratio was significantly lower in males than in females (24.3 vs 25.7, *P* < .001) (Fig. 3).

**Figure 4:**
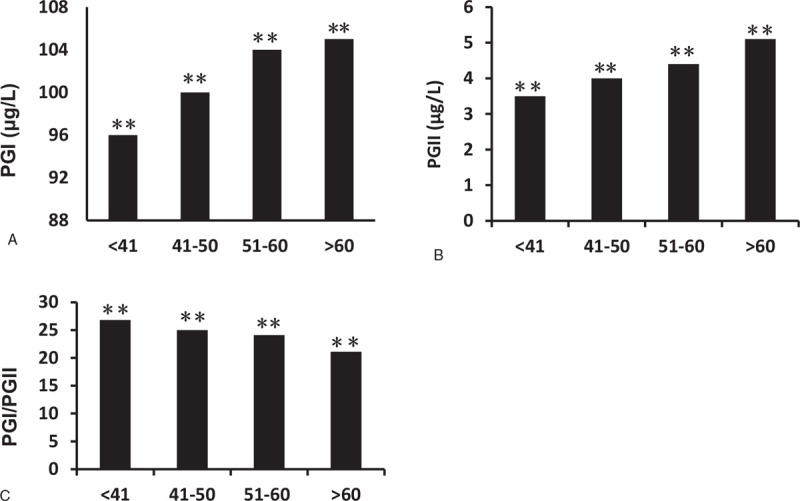
Serum PGI, PGII levels, and PGI/PGII ratio in different ages. All subjects have been divided into 4 groups, including <41 years’ old group, 41 to 50 years’ old group, 51 to 60 years’ old group, and >60 years’ old group. As age increases, both PGI and PGII level increases (A and B), PGI/PGII ratio decreases (C). Data are means ± SEM. ^∗∗^*P* < .01 comparison among all age groups. PG = pepsinogen.

We also compared the PG1, PGII, and PGI/PGII levels from inclusive subjects stratified by age: they are: <41-year-old group (n = 13,216), 41- to 50-year-old group (n = 9680), 51- to 60-year-old group (n = 10,184), and >60-year-old group (n = 7303). As shown in Figure 4, the serum PGI levels were 96 (75–122) ng/mL, 100 (80–128) ng/mL, 104 (81–133) ng/mL, and 105 (82–137) ng/mL from the younger to older groups. The serum PGII levels were 3.5 (2.0–6.1) ng/mL, 4.0, (2.2–6.9) ng/mL, 4.4 (2.4–7.7) ng/mL, and 5.1 (2.6–8.9) ng/mL from the younger to older groups (*P* *<* .001). The PGI/PGII ratio decreased with aging (27, 25, 24, and 21 respectively, *P* < .001) (Fig. 4).

### 3.2 The relationship between PG levels and BMI

All the inclusive subjects were assigned to 4 groups based on their BMI (body weight [BW] [kilograms]/height [square meters]): group A (<18.5, low BW), group B (>18.5–24.0, normal BW), group C (>24.0–28.0, overweight), and group D (>28, obese). The serum PG levels were compared between different groups. As shown in Table 2, the serum PGI level in groups A, B, and C remained relatively stable, there was no significant difference among groups (*P* > .05). BMI >28 subjects had lower serum PGI level than subjects in the other 3 groups. Similar to the serum PGI level, PGII also remained stable in groups A, B and C, and the serum PGII level was significantly lower in obese subjects (all *P* < .05) (Table 2). There was a difference of distribution in the ratio of PGI/II. In group D, subjects showed a higher ratio than the other 3 groups (all *P* < .05) (Table 2).

**Table 2:** Serum PG levels in different BMI (median [IQR 25%–75%]).

Group (BMI kg/CM)	Cases	PGI, ng/mL	PGII, ng/mL	PGI/PGII
A (<18.5)	671	100 (78–127)	4.5 (2.5–7.5)	23
B (>18.5–24)	11,805	100 (78–129)	4.1 (2.3–7.3)	24
C (>24–28)	14,421	100 (79–129)	4.2 (2.3–7.4)	24
D (>28)	8146	97 (77–124)^∗^	3.9 (2.2–6.8)^∗^	25^∗^

BMI = body mass index, IQR = interquartile range, PG = pepsinogen I, PGII = pepsinogen II.

∗ *P* < .05 vs group A, B, and C.

## 4 Discussion

In this study, we measured and analyzed the serum PG levels and compared with Hp status in the largest sample size of Chinese subjects to this date. Our results showed that Hp infection had a positive correlation with PGI and PGII, and a negative correlation with PGI/PGII. In fact, PGII had doubled in the asymptomatic Hp infected population. Our results were in agreement with other studies: Hp infection influences the serum PG level, and the serum PG levels in Hp+ individuals were significantly higher than those in Hp− individuals, and the PGI/PGII ratio was lower in Hp+ individuals than in Hp− individuals, confirming.^[11,16,17]^ In this study, however, we found a much higher PGII level increase than in other studies. The reason remains to be explored. The mechanism of Hp infection causing an increase in PGs may be linked to Hp stimulating antral G-cells, thus increasing the level of gastrin and further promoting PG secretion.^[22]^ The combination of pepsinogen, gastrin-17 and anti-Hp antibodies serological assays seemed to be a reliable tool for the diagnosis of atrophic gastritis.^[9]^ There was study shown that Chinese patients with higher PGII levels were at greater risk of various Hp-related gastropathies.^[23]^ Recently, Lee^[24]^ reviewed the association between the serum pepsinogen levels and Hp infection and found pepsinogen levels are linked to endoscopic gastritis and Hp infection. The accumulated gastric cancer incidence in the subjects with abnormal PG levels and those with Hp infection were all significantly higher than that in normal controls. Both abnormal serum PG level and Hp infection were risk factors for the development of gastric cancer.^[11]^

Our results also suggest that sex impacts serum PG levels, as males have higher serum PGI and PGII levels than females. These results agree with other studies on the Chinese population.^[16,17]^ The same result was reported by Hokkanen et al in Japanese patients,^[25]^ as well as Pals et al’ report in healthy European subjects.^[26]^ Thus, it would be appropriate to apply different threshold values for different sexes when reporting serum PG levels. The usefulness of PGI/PGII ratio is less clear, since we found a lower ratio in males than females, which is not consistent with others report.^[16,17]^

Age is an important factor which affects many physiological and pathological processes of the human body. Our results showed that both serum PGI and PGII levels increased with age, while the ratio of PGI/PGII decreased with age because of a greater extent of PGII level increase. This finding was in agreement with Huang's study.^[17]^ Kitahara et al^[12]^ also reported PGII level increased and PG I/II level decreased with progression of age, whereas Sun et al's study showed that serum PGI levels decreased in persons older than 61 years.^[16]^ In European studies, one showed that the serum PGI and PGII levels increased with advancing age in a healthy population,^[27]^ another study showed levels of PGI and the PG I/II ratio decreased with ageing in Hp infected subjects.^[28]^ The evidence from our study along with others showed that age was an influencing factor for serum PG levels, although there were conflicting results. Further investigations are recommended to identify its normal distribution in different age groups through stratification.

Our results showed that in obese subjects (group D, BMI >28) serum PGI and PGII levels were lower than other categories, whereas PGI/PGII ratio was higher in this category. These results were in partial agreement with Kutsuma et al finding that serum PGI, but not PGII or PGI/PGII, was significantly reduced across the increasing BMI categories.^[29]^ It has reported that PGI/II ratio had positive correlation with BMI, that is, lower BMI subjects had lower PGI/II.^[30,31]^ When using PGs to diagnose atrophic gastritis and to analyze the relationship between Hp infection and BMI, it found that BMI was associated with PG levels, but not with Hp infection status.^[30]^ Studies have shown the trend of higher BMI in patients with gastric cardia cancer.^[13,15]^ The effect of obesity on gastric cancer showed sex differences. In men it was related to increased risk of early gastric cancer and well or moderately differentiated adenocarcinoma, but it was associated with gastric dysplasia in women regardless of Hp infection in Korea.^[32]^ It was still unclear whether the changes in serum PGI and PGII were relevant to pathogenesis of obesity-related diseases.

There were limitations in this study. First, we included asymptomatic subjects in this study; however, these subjects were not experienced gastroendoscopy and pathology, so some asymptomatic patients with CAG or other gastric diseases could not be excluded from this study. Therefore, our result could not reflect the accuracy of the stomach. Second, this study did not deeply analyze the relationship of between BMI, age and the risk of Hp infection; hence, it is worthy for further study. Third, follow-up of the subjects in this study would be useful to provide further insight into the relationship of PGI/PGII secretion with Hp infection, sex, age, and BMI, especially the influence of eradication of Hp on the serum PG levels and PGI/PGII ratio.

In conclusion, we analyzed the serum PG levels and PGI/PGII ratio on a large sample of Chinese population and the results showed that Hp infection increased PGII by 2-fold, and PGs were associated with sex, age, and BMI. Our results might be a useful reference for clinical practice and further research on the roles of PGs in various PG and Hp-related diseases.

## Author contributions

**Conceptualization:** Fong-Fong Chu, Qiang Gao.

**Data curation:** Hong Yu, Ying Liu, Shujing Jiang, Yunfeng Zhou, Siyuan Dong, Fong-Fong Chu, Chunbo Kang, Qiang Gao.

**Formal analysis:** Hong Yu, Yunfeng Zhou, Fong-Fong Chu.

**Funding acquisition:** Ying Liu, Yunfeng Zhou, Qiang Gao.

**Investigation:** Hong Yu, Ying Liu, Shujing Jiang, Chunbo Kang.

**Methodology:** Zheng Guan, Siyuan Dong.

**Project administration:** Ying Liu.

**Software:** Yunfeng Zhou, Zheng Guan, Siyuan Dong.

**Supervision:** Ying Liu, Chunbo Kang, Qiang Gao.

**Visualization:** Zheng Guan, Siyuan Dong.

**Writing – original draft:** Shujing Jiang, Fong-Fong Chu, Qiang Gao.

**Writing – review & editing:** Shujing Jiang, Fong-Fong Chu, Qiang Gao.
